# Overexpression of E2F mRNAs Associated with Gastric Cancer Progression Identified by the Transcription Factor and miRNA Co-Regulatory Network Analysis

**DOI:** 10.1371/journal.pone.0116979

**Published:** 2015-02-03

**Authors:** XiaoTian Zhang, ZhaoHui Ni, ZiPeng Duan, ZhuoYuan Xin, HuaiDong Wang, JiaYi Tan, GuoQing Wang, Fan Li

**Affiliations:** Department of Pathogenobiology, The Key Laboratory of Zoonosis, Chinese Ministry of Education, College of Basic Medicine, Jilin University, Changchun, Jilin, China

## Abstract

Gene expression is regulated at the transcription and translation levels; thus, both transcription factors (TFs) and microRNAs (miRNA) play roles in regulation of gene expression. This study profiled differentially expressed mRNAs and miRNAs in gastric cancer tissues to construct a TF and miRNA co-regulatory network in order to identify altered genes in gastric cancer progression. A total of 70 cases gastric cancer and paired adjacent normal tissues were subjected to cDNA and miRNA microarray analyses. We obtained 887 up-regulated and 93 down-regulated genes and 41 down-regulated and 4 up-regulated miRNAs in gastric cancer tissues. Using the Transcriptional Regulatory Element Database, we obtained 105 genes that are regulated by the E2F family of genes and using Targetscan, miRanda, miRDB and miRWalk tools, we predicted potential targeting genes of these 45 miRNAs. We then built up the E2F-related TF and miRNA co-regulatory gene network and identified 9 hub-genes. Furthermore, we found that levels of E2F1, 2, 3, 4, 5, and 7 mRNAs associated with gastric cancer cell invasion capacity, and has associated with tumor differentiation. These data showed Overexpression of E2F mRNAs associated with gastric cancer progression.

## Introduction

Gastric cancer is still one of the most significant health problems in developing countries, like in China, although its incidence is gradually decreasing in the Western countries. Overall, gastric cancer is accounts for the fourth of incidence and the second of mortality rates among all cancers in the world [1–3]. Gastric cancer risk factors include Helicobacter pylori infection, frequent consumption of smoked foods, salted fish and meat, and pickled vegetables, tobacco smoke, obesity, or chronic gastritis. These risk factors could coordinate to manipulate gene expression or mutation or epigenetic alterations and eventually result in gastric cancer development. To date, a large body of knowledge has accumulated regarding the molecular alterations associated with gastric cancer, such as ARID1A, TP53 [4], PTGER4, PRKAA1, ZBTB20 [5] and PLCE1 [6]. However, the underlying mechanism for different genes-mediated gastric carcinogenesis remains to be defined. Thus, it is crucial to further investigate molecular pathogenesis of gastric cancer using the systematical biology approach, such as the construction of differentially expressed genes-regulatory network to identify the important gene pathway or signaling during gastric cancer development or progression.

Gene expression is regulated at the transcription and translation levels. At the transcription level, gene transcription factors (TFs) play an important role in regulation of human gene expression, while miRNA could at the post-transcription level regulate mRNA translation and half-life. Specifically, TFs are proteins that bind to specific DNA sequences and thereby control gene transcription. MiRNA is a class of naturally occurring small noncoding RNAs with 18 to 22 nucleotides in length and functionally, miRNA can post-transcriptionally silence protein expression by binding to complementary target gene transcripts, thereby degrading these messenger RNAs or inhibiting them from translating into proteins. Thus, both TFs and miRNAs can regulate genes at different stages of gene expression and may form a feedback loop and a complicated regulatory network to tightly control gene expression. In this regard, study of this gene regulatory network could help us to understand cell homeostasis and physiological process, biological function, and mechanism of diseases. To date, a number of studies have shown gene regulation of TFs and miRNA in gastric cancer, such as nuclear factor kappa B [7], FoxM1 [8], hypoxia-inducible factor 1 [9], and miR-7 [10], miR-375 [11], miR-125b, miR-199a, miR-100 [12]. Indeed, aberrant miRNA or TF expression contributes to human carcinogenesis [13]. Therefore, in this study, we investigated the role of the combined miRNA and transcription factors in regulation of gene expression in gastric cancer for association with gastric cancer progression. We first detected differential expression of genes and miRNAs in gastric cancer tissue samples and analyzed them bioinformatically to form the TF-miRNA regulatory network to relate expression of E2F family mRNAs in gastric cancer. We then confirmed E2F expression for association with gastric cancer progression.

## Materials and Methods

### Patients and tissue specimens

This study was approved by the Ethics Committee of School of Basic Medical Sciences, Jilin University and each patient was consented in a written informed consent form. After that, we enrolled 70 gastric cancer patients from Jilin University (Changchun, China) between April 2012 and October 2014. All patients did receive any pre-surgery treatment, like chemo- or radiotherapy. Both tumor and distant normal tissues were obtained from the operation room and stored in liquid nitrogen within 10 min.

### RNA isolation and miRNA preparation

Total cellular RNA was isolated from tissue specimens using the Trizol reagent (Invitrogen, Carlsbad, CA, USA) and then further purified using an RNeasy Mini kit (Qiagen, Düsseldorf, Germany). RNA concentration was then determined using the Epoch Multi-volume Spectrophotometer System (BioTek, Vermont, USA). After that, miRNA was isolated from these RNA samples using the mirVana miRNA isolation Kit (Ambion, Austin, TX, USA).

### Exon microarray analysis

In this study, we first profiled gene expression between 45 gastric cancer and paired adjacent normal tissue samples using the Affymatrix Gene Chip Exon Arrays 1.0 ST (Affymatrix, CA, USA). Specifically, 1μg RNA sample was reversely transcribed into cDNA and these cDNA samples were then digested into cDNA fragments with endonucleases and labeled with the DNA labeling reagent using DNA Labeling Kit (Affymatrix, CA, USA). The labeled cDNA templates were used as probes to hybridize to the Affymatrix Gene Chip Exon Arrays 1.0 ST in a condition of 45°C incubation and rotation at 60 rpm for 17 h. After that, the arrays were washed and scanned using Gene Chip Scanner 3000 with Gene Chip Operating Software (GCOS).

### MiRNA microarray analysis

We also profiled the differentially expressed miRNAs in 15 gastric cancer and paired adjacent normal tissue specimens using Affymatrix Gene Chip microRNA array. Similar to the cDNA microarray experiments, the miRNA probes using RNA Labeling Kit (Affymatrix, CA, USA) were hybridized to Affymatrix Gene Chip microRNA array at 45°C and rotated at 60 rpm for 17 h. After that, the arrays were scanned using GCOS.

### Microarray data analysis

The raw microarray data were analyzed using Limma algorithm to identify the differentially expressed genes and miRNAs and then analyzed using the linear models and empirical Bayes methods. A *t*-test and Bonferroni correction was used for assessing the statistical significance of each differential expression. Genes and miRNA were considered to be significantly differentially expressed if *p*-values< 0.05 and gene expression showed at least 1.5-fold changes between cancer and their normal tissues. QUBIC (Qualitative BI-Clustering) program was utilized to cluster-analyze differentially expressed genes. The basic idea of the algorithm is to find all subgroups of genes with similar expression patterns among some subsets of cancer tissues, and hence genes involved in each such pattern can possibly be used as signatures for cancer sub-typing or staging. For our bi-cluster analysis, we have used the following parameters: r = 1, q = 0.06, c = 0.95, o = 100, f = 1 [14,15]. Database for Annotation, Visualization and Integrated Discovery (DAVID) and Kyoto Encyclopedia of Genes and Genomes (KEGG) tools were used for functional analysis and pathway classification of these different genes.

### qRT-PCR

Total cellular RNA from cancerous and normal gastric tissues were reversely transcribed into cDNA using 1^st^ strand cDNA Synthsis Kit (Takara, Dalian, China) according to the manufacturer’s recommendations. Expression of E2F1, E2F2, and E2F4) mRNA was analyzed in 10 gastric cancer and paired adjacent normal tissue specimens by q-PCR using SYBR Premix Ex Taq (Takara) and β-actin was used as an internal control. The primers are listed in Table 1. The qPCR data were for quantified by using 2^ΔΔCt^ methods.

**Table 1:** Primers used for qPCR.

Gene	Forward	Reverse
β-Actin	5’- CTGGAACGGTGAAGGTGACA-3’	5’- AAGGGACTTCCTGTAACAATGCA-3’
E2F1	5’- CATCCCAGGAGGTCACTTCTG-3’	5’- GACAACAGCGGTTCTTGCTC-3’
E2F2	5’- CGTCCCTGAGTTCCCAACC-3’	5’- GCGAAGTGTCATACCGAGTCTT-3’
E2F4	5’- ATCGGGCTAATCGAGAAAAAGTC-3’	5’- TGCTGGTCTAGTTCTTGCTCC-3’

### Construction of the TF-miRNA co-regulatory network

After microarray data analysis, we obtained TFs and the latent target genes that are regulated by the E2F family through searching of Transcriptional Regulatory Element Database (TRED). Moreover, the miRNAs targeting E2F family was determined interrogating four target prediction databases, i.e., Targetscan, miRanda, miRDB, and miRWalk database [16–18]. We then combined these differential expressed genes and miRNAs to construct this TF-miRNA co-regulatory network related to E2F family using Cytoscape software. In the TF-miRNA co-regulatory network, we defined node as a hub gene or miRNA when it directly linked to more than two total nodes in the network.

### Analysis of E2F family mRNAs for association with clinic pathological characteristics from gastric cancer patients

Using the receiver operating characteristic (ROC) curves, we analyzed differentially expressed genes that are regulated by the E2F mRNAs between gastric cancer and paired adjacent normal tissues gene. We then selected and distinguished the best matched genes for association with clinic pathological characteristics using the binary logistic regression analysis.

### Statistical analysis

The ROC curve and binary logistic regression analysis were utilized for differentially expressed genes that are regulated by the E2F mRNAs between gastric cancer and paired adjacent normal tissues. One-way ANOVA was utilized to associate between E2F family and degree of tumor cell invasion. GraphPad Prism 6 software was performed to obtain ROC curve and calculation of sensitivity, specificity and area under the curve (AUC). SPSS 18.0 software was used for One-way ANOVA and binary logistic regression analysis. A p value <0.05 was considered to be statistically significant.

## Results

### Detection of differentially expressed genes and miRNAs between gastric cancer and their corresponding normal tissues

**Fig 1:**
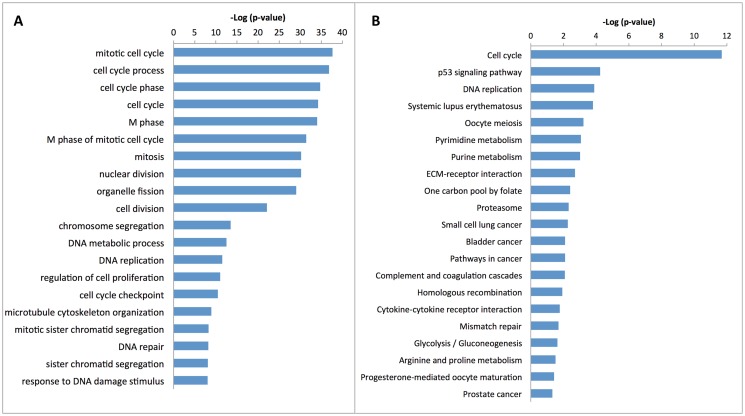
Significant pathways of 980 differentially expressed genes. A, Gene ontology analysis; B, KEGG analysis.

We first performed the Affymatrix Exon Arrays analysis to detect differentially expressed genes between gastric cancer and paired adjacent normal tissue in 45 patients (patients’ data are shown in S1 Table). The GEO Datasets of NCBI accession number of this study was GSE63089. Using> 1.5 fold changes as the cut-off-value, we identified 887 up-regulated and 93 down-regulated genes (S2 Table). Functional analysis showed that these differential genes mainly formed gene pathways, such as cell cycle control, p53 signaling pathway, cancer pathway, extracellular matrix-receptor interaction, cell adhesion, glycolysis/gluconeogenesis, and cytokine receptor interaction (Fig. 1). E2F family members play a major role during cell cycle G1/S transition in cells and the gene expression of E2F1, 2, 3, 4, 5, and 7 were all found to be overexpression (p < 0.01) in gastric cancer in this study.

**Fig 2:**
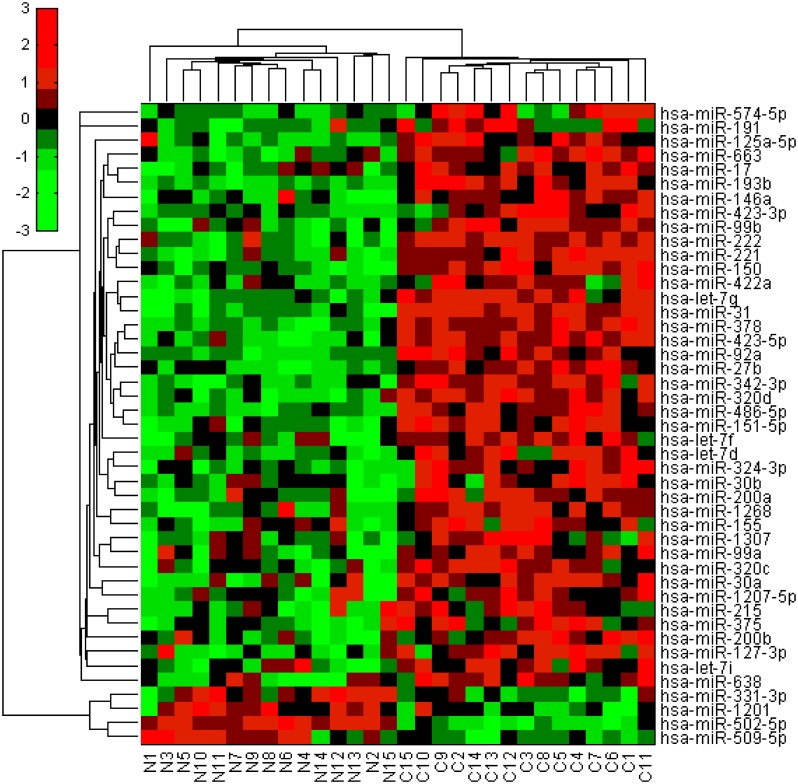
Bi-clusters analysis of 45 differentially expressed miRNAs in gastric cancer vs. normal tissues. Each row represents a miRNA and each column represents a sample. The “C” columns at the bottom represent cancer tissues, while “N” represents normal tissues. Red stands for high expression in cancer compared to normal tissue, while green for low expression in cancer compared to normal tissues.

Next, we also performed the Affymatrix Gene Chip microRNA array analysis in 15 cases of gastric cancer and paired adjacent normal tissues (patients’ data are shown in S1 Table). The GEO Datasets of NCBI accession number of this study was GSE63121. We found 41 down-regulated and 4 up-regulated miRNAs (S3 Table). Fig. 2 illustrated the bi-clusters cluster analysis of those 45 differential miRNAs in gastric cancer vs. normal tissues. Functionally, these differentially expressed miRNAs could regulate different gene pathways, such as transcription activator activity, DNA binding, transcription factor activity, post transcriptional regulation of gene expression, and G1/S transition of mitotic cell cycle.

### Building-up and analysis of the E2F family-related TF-miRNA co-regulatory network

**Fig 3:**
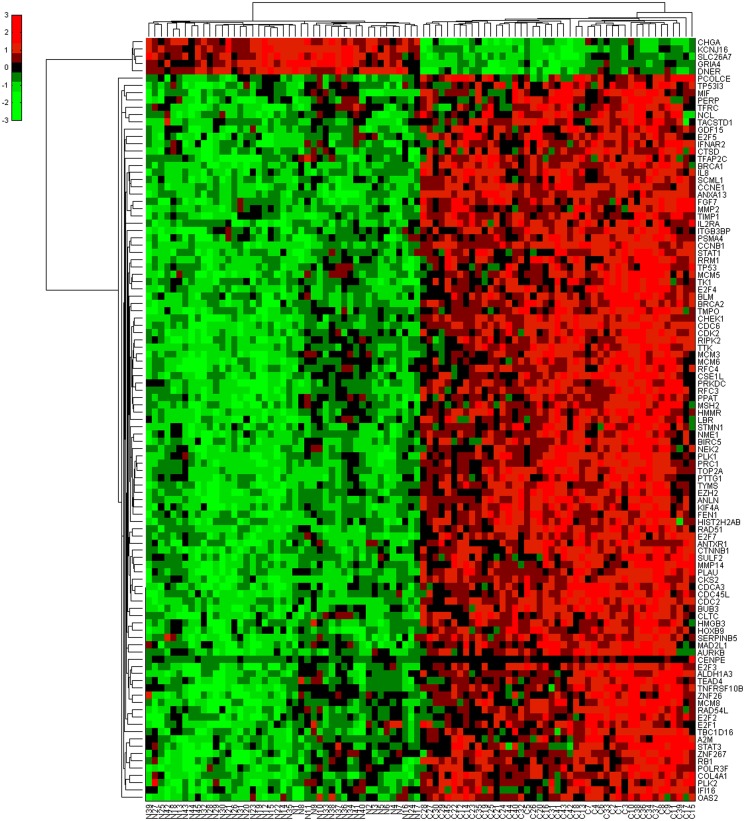
Bi-clusters analysis of 105 differentially expressed genes in TF-gene regulatory network. Each row represents a miRNA and each column represents a sample. The “C” columns at the bottom represent cancer tissues, while “N” represents normal tissues. Red stands for high expression in cancer compared to normal tissue, while green for low expression in cancer compared to normal tissues.

**Fig 4:**
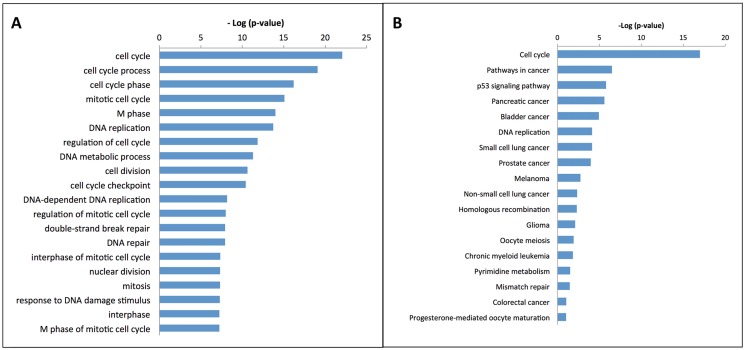
Significant pathways of 105 differentially expressed genes regulated by TF-gene regulatory network. A, Gene ontology analysis; B, KEGG analysis.

In order to display the E2F family-related TF-miRNA co-regulatory network, we utilized TRED to inquire TF and latent target genes regulated by E2F family and then selected the differentially expressed TFs and latent target genes in gastric cancer tissues. We found a total of 105 TFs and latent target genes that could be potentially regulated by E2F family in gastric cancer (5 down-regulated and 100 up-regulated genes; see S4 Table), which could form a 105 gene network after the bi-clusters cluster analysis (Fig. 3) The DAVID analysis showed these 105 genes are most cell cycle-related genes (Fig. 4).

**Fig 5:**
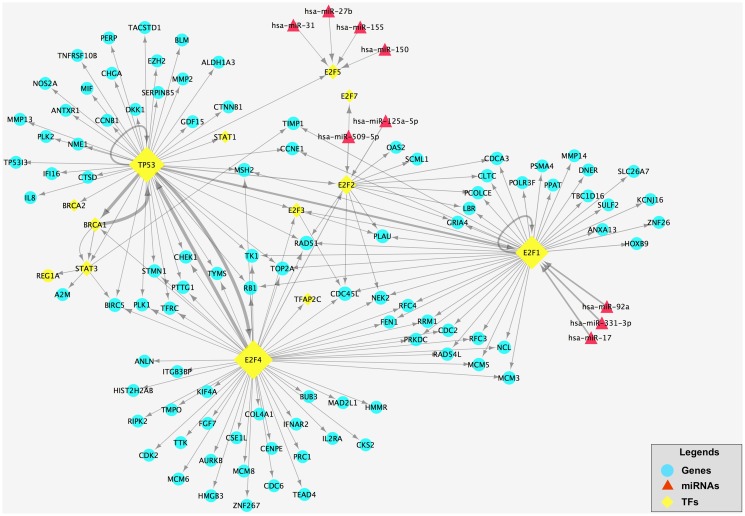
The E2F-related TF-miRNA network in gastric cancer tissues. This network contains 105 differentially expressed genes and 9 differentially expressed miRNAs. Blue circles are differentially expressed genes and the red diamonds are differentially expressed miRNAs. The yellow rhombuses are TFs and its size represents the numbers of target genes. The direction of the arrow is from the source to the target.

Next, we utilized online tools Targetscan, miRanda, miRDB and miRWalk database to predict potential targeting genes of these 45 miRNA and then merged these targeting genes with these 105 differentially expressed genes. We found 7 down-regulated and 2 up-regulated miRNAs (S3 Table). After that, we built up the E2F-related TF-miRNA regulatory network (Fig. 5).

**Fig 6:**
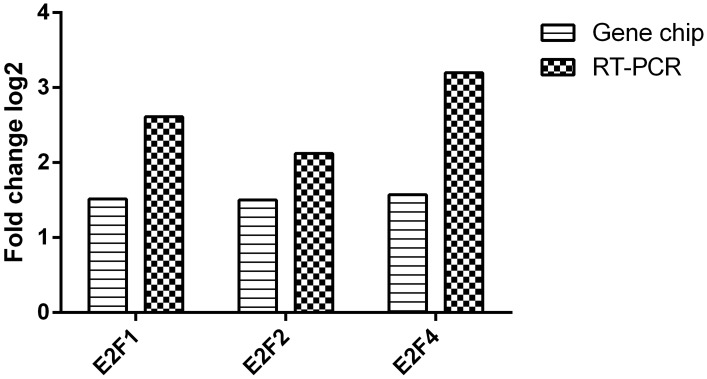
Validation of differential expression of mRNAs using qRT-PCR. Gene expression was analyzed in 10 paired gastric cancerous and normal tissues and compared to microarray results. The columns in the chart represent the log-transformed median fold changes (cancer/control) in expression across ten samples (p<0.05).

**Fig 7:**
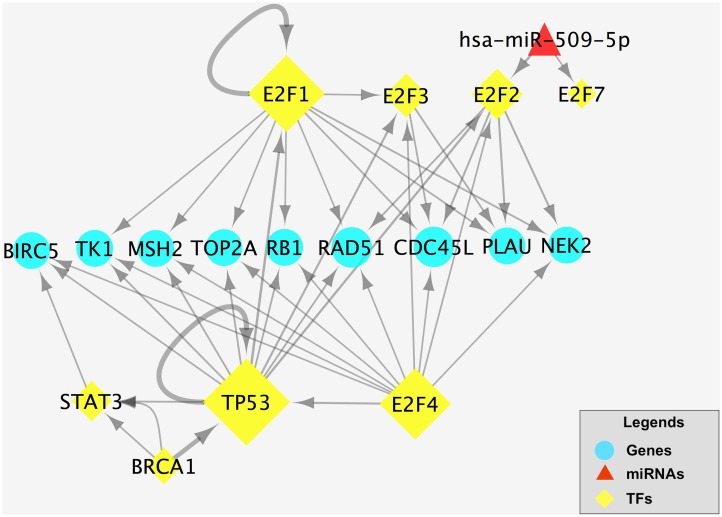
Detection of the hub genes based on The E2F-related TF-miRNA network analysis. Circles are hub genes that co-regulated by TFs and miRNAs, while the red diamonds are differentially expressed miRNAs. The yellow rhombuses are TFs and its size represents the numbers of target genes. The direction of the arrow is from the source to the target.

After that, we analyzed this E2F-related TF-miRNA regulatory network and found that E2F1, E2F2 and E2F4 in E2F family play an important role in this TF and miRNA co-regulatory network. Three over-regulated mRNAs (E2F1, E2F2 and E2F4) from the differentially expressed mRNAs were validated using real-time PCR (RT-PCR). The results demonstrated that E2F1, E2F2 and E2F4 were overregulated in the gastric cancer samples compared with normal samples. The RT-PCR results and microarray data are consistent (p< 0.05, Fig. 6). In addition, we identified 9 hub-genes from E2F-related TF-miRNA regulatory network, which can be co-regulated by TFs (Fig. 7). The DAVID analysis of these 9 hub genes and functions is shown in Table 2.

**Table 2:** Significant GO pathways of the hub-genes regulated by the TF-miRNA co-regulatory network.

Term	p value	Genes	Fold Enrichment	Benjamin
GO:0007049, cell cycle	1.34E-05	NEK2, MSH2, BIRC5, RB1, RAD51	24.48	0.002
GO:0022402, cell cycle process	1.97E-04	NEK2, MSH2, BIRC5, RAD51	27.35	0.019
GO:0051276, chromosome organization	5.61E-04	NEK2, MSH2, RB1, TOP2A	19.20	0.037

### Association of E2F family mRNA levels with clinic pathological characteristics from gastric cancer patients

**Fig 8:**
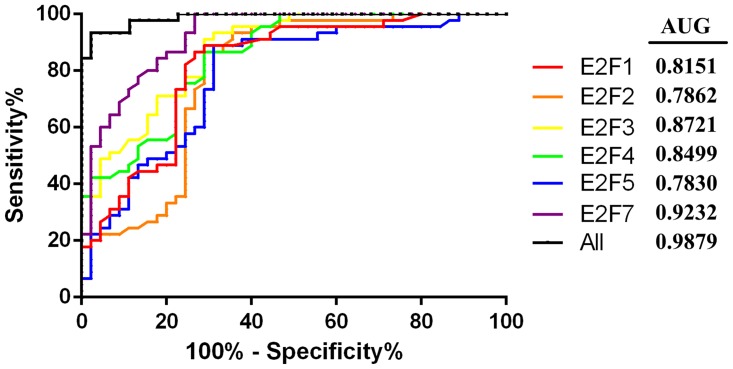
ROC curve as discriminators between cancer and normal tissues. AUC, Area under the ROC Curve; All indicates the combination of E2F1, E2F2, E2F3, E2F4, E2F5, and E2F7.

**Fig 9:**
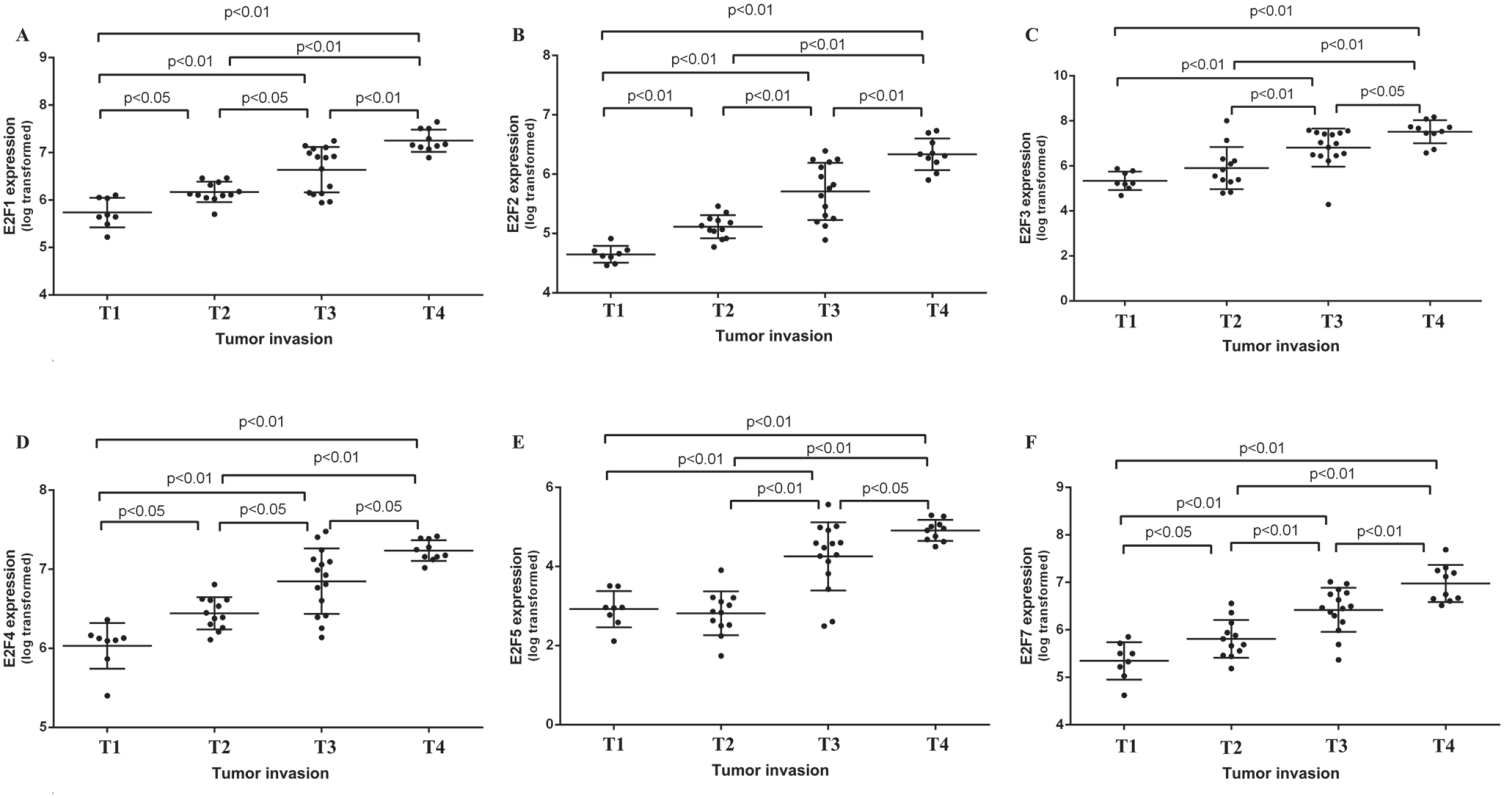
Expression of E2F1, E2F2, E2F3, E2F4, E2F5, and E2F7 mRNAs associated with the depth of gastric cancer invasion. Microarray data from 45 gastric cancer cases were used to analyze association between E2F family and gastric cancer invasion. Gastric cancer invasion was referred to the International Union against Cancer (UICC) TNM staging system. T1, Tumor invades lamina propria, muscularis mucosae, or submucosa; T2, Tumor invades muscularispropria; T3, Tumor penetrates subserosal connective tissue without invasion of visceral peritoneum or adjacent structures; T4, Tumor invades serosa (visceral peritoneum) or adjacent structures.

**Fig 10:**
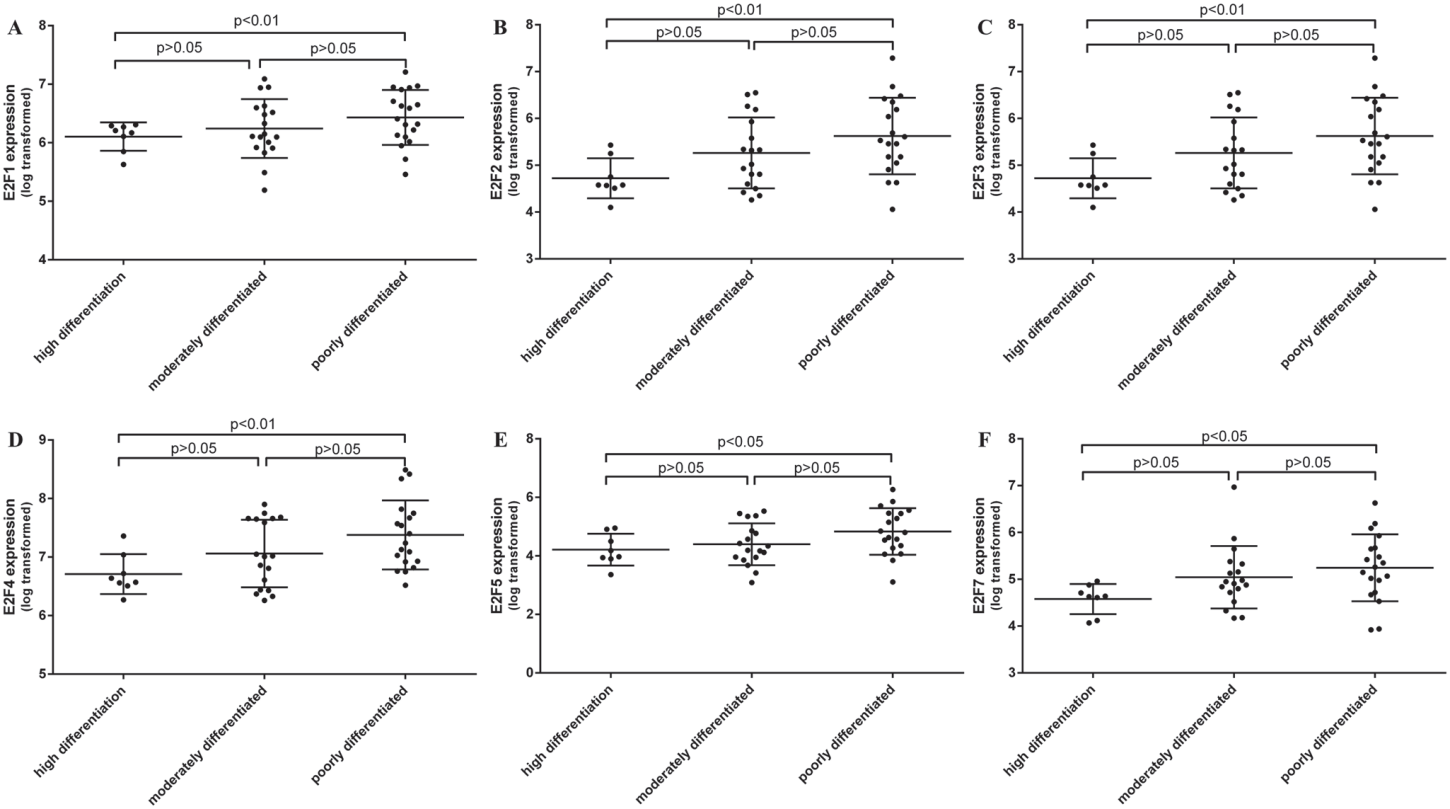
Expression of E2F1, E2F2, E2F3, E2F4, E2F5, and E2F7 mRNAs associated with gastric cancer differentiation.

We further analyzed expression of E2F mRNAs and then associated them with clinic pathological characteristics from gastric cancer patients. The ROC curves analysis showed that E2F1, 2, 3, 4, 5, and 7 can be the latent targets to distinguish gastric cancer tissue and the normal ones (Fig. 8). Combination of several E2F family mRNAs can further improve the specificity and sensitivity of their distinguishing between gastric cancer and normal tissues after the regression of binary logistic analysis (Fig. 8). Moreover, we found level of E2F1, 2, 3, 4, 5, and 7 mRNA associated with depth of gastric cancer invasion (Fig. 9). We also found that E2F expression has associated with tumor differentiation (Fig. 10).

## Discussion

In this study, we utilized a cut-off value of 1.5 fold change to profiled differentially expressed mRNAs and miRNAs in gastric cancer tissues, which is consistent with most of previous study of cDNA or miRNA microarrays [19]. These differentially expressed mRNAs and miRNAs in gastric cancer tissues were mostly related to cell cycle progression, especially E2F family. To date, the members of E2F proteins include E2F1- E2F8 and among of them, E2F1, 2, 3, 4, 5, and 7 proteins were all significantly overexpressed in gastric cancer in the current study. Thus, we predicted that E2F family has important regulatory functions in gastric cancer. Thus, we constructed this E2F-related TF-miRNA co-regulatory network for gastric cancer based on our microarray profiling data. This network contains 105 TFs and their regulated latent target genes (5 down-regulated and 100 up-regulated genes) that are related to E2F family and 9 differential miRNAs (7 down-regulated and 2 up-regulated miRNAs) (Fig. 5). In other words, E2F family of genes could act on these 105 genes and 9 miRNAs to regulate cell cycle progression of gastric cancer. Indeed, we found that target genes regulated by E2F1 and E2F4 appeared quantities of differential expression in gastric cancer, which indicates that E2F1 and E2F4 are very likely to occupy an important share in growth of gastric cancer. Furthermore, miRNAs differentially expressed in gastric cancer were able to regulate expression of E2F1, E2F2, E2F5 and E2F7, indicating that miRNAs-altered expression of E2Fs proteins is important factors in gastric cancer development or progression.

Indeed, E2F family members play a major role during cell cycle G1/S transition in cells and their altered expression contributed a number of human diseases, including cancer [20]. For example, during gastric cancer growth and progression, cancer cells will promote tumor cell proliferation, but inhibit apoptosis. At the gene level, transcription factor E2F family of genes plays a significant role in regulating cell cycle process by promoting the timely expression of genes required for DNA synthesis at the G1/S phase transition. E2F activity itself is controlled by retinoblastoma protein (RB) and the pocket proteins p107 and p130. To date, the members in E2F proteins are E2F1–E2F8, including transcription-activated factors E2F1–3a, which mainly regulate cell cycle transition from G0 to S phase, and transcription inhibited factors E2F3b-E2F8, which are expressed in quiescent or differentiated cells and prevent cell cycle progression [21]. Previous studies demonstrated that altered expression of E2F gene family was closely associated with growth of breast cancer [22], ovarian cancer [23], bladder cancer [24], colorectal cancer and pancreatic cancer [25]. In gastric cancer, previous studies showed that E2F was abnormally expressed and E2F expression regulated by miRNAs was associated with cell cycle progression and apoptosis repression in gastric cancer cells to suppress TGFβ tumor suppressor pathway [26]. E2F gene mutation is also one of the causes of early gastric cancer occurrence [27]. Our current data are supported this finding.

However, besides E2F family, TP53, BRCA1, and STAT3 also play an important role in this TF and miRNA co-regulatory network (Fig. 5). For example, TP53is one of the most widely studied genes and plays a role in regulation of apoptosis, genomic stability, and angiogenesis [28]. A previous study showed that *p53* mutation directly linked to development of gastric cancer [4] and another study showed that restoration of p53 activity induced the sensitivity of gastric cancer to chemotherapy [29]. BRCA1 is the susceptibility gene of breast cancer and regulates cell apoptosis and repairs DNA damage. BRCA1 plays a role in regulation DNA repair activity and *BRCA1*mutation contributed to development of breast cancer, ovarian cancer [30], pancreatic cancer [31], and gastric cancer [32]. Lost expression of BRCA1 protein was associated with poor survival rate of gastric cancer patients [33]. Our current study showed that BRCA1 mRNA was associated with gastric cancer differentiation. Moreover, STAT3 is a transcription factor that is activated in response to growth factors and cytokine, and contributes to regulation of cell proliferation, apoptosis, and motility in cells. Previous studies showed that STAT3 was a key regulatory factor [34] in gastric cancer development and that STAT3 activation promoted tumor cell survival and migration [35]. A previous study utilized STAT3 inhibitor to treat gastric cancer and showed efficacy [36].

Furthermore, miRNAs (miR-125a-5p, miR-331–3p, miR-17, miR-150, miR-155, miR-27b, miR-31, miR-92a and miR-509–5p), which differentially expressed in gastric cancer, were found to regulate expression of E2F1, E2F2, E2F5 and E2F7 in this study (S3 Table). Previous studies showed that miR-125a-5p expression was associated with gastric carcinogenesis by targeting of E2F3 [37]. MiRNA-331–3p directly targets E2F1 and induced growth arrest of human gastric cancer cells [38]. Moreover, expression of miR-155 was able to block TGF-β1-mediated activation of the Rb and in turn to decrease the abundance of the inhibitory pRB-E2F1 complex and lift G0/G1 arrest [39]. MiR-17 family clusters are emerging as key modulators of TGF-βtumor suppressor signaling in gastric cancer, through regulation of p21, E2F1–3 and E2F5 target gene expression [40–42]. In addition, miR-150, miR-31, and miR-92a were also shown closely related to gastric cancer [43–46]. Although there have been no reported about miR-509–5p related to gastric cancer, miR-509–5p joined the Mdm2/p53 feedback loop and regulates cancer cell growth [47]. These studies were consistent with our current results.

In addition, the regulatory relationship between TF-genes and miRNA-TF may have the synergistic effects. Based on our newly established E2F-related TF-miRNA co-regulatory network, we identified 9 hub-genes that mainly involve in cell cycle and chromosome organization (Fig. 7). Taken all data together, our current study indicate that gastric cancer development and progression are involved multiple genes and further studies will focus on these genes as novel targets for control of gastric cancer.

However, our current study just provided a preliminary data and further confirmation study is needed to verify our data in ex vivo and in vitro. This systematical approach may help us to explore cancer pathogenesis and provide the theoretical basis for searching novel strategy for treatment of gastric cancer in future.

## Supporting Information

S1 Table Characteristics of patients. (DOC)

S2 Table Summary of 980 differentially expressed genes in gastric cancer tissues compared to the distant normal tissues. Gene expression levels in gastric cancer tissues vs. the distant normal tissues were at least 1.5-fold different with a p-value <0.05.
(XLSX)

S3 Table Summary of 45 differentially expressed miRNAs in gastric cancer tissues compared to the distant normal tissues. Levels of miRNA expression in gastric cancer tissues vs. the distant normal tissues were at least 1.5-fold different with a p-value <0.05.
(XLSX)

S4 Table Summary of 105 differentially expressed genes in the TFs-regulatory network in gastric cancer tissues. (DOCX)
