# Supplementary material for: Overexpression of E2F mRNAs Associated with Gastric Cancer Progression Identified by the Transcription Factor and miRNA Co-Regulatory Network Analysis
Source: PLoS One. 2015 Feb 3;10(2):e0116979. doi: 10.1371/journal.pone.0116979 (PMC4315469; doi:10.1371/journal.pone.0116979)
Supplement: S1 Table — (DOC) [file pone.0116979.s001.doc]

Table S1. Clinicopathological characteristics of the study samples

| Case | Participate | Clinical parameters | | | |
| --- | --- | --- | --- | --- | --- |
|  |  | ***Gender*** | ***Age*** | ***Differentiation status*** | ***T stage*** |
| 1 | microarray | Female | 53 | Poorly differentiated | T3 |
| 2 | microarray | Female | 54 | Moderately differentiated | T3 |
| 3 | microarray | Female | 62 | Poorly differentiated | T4a |
| 4 | microarray | Male | 63 | Poorly differentiated | T3 |
| 5 | microarray | Male | 56 | Poorly differentiated | T4a |
| 6 | microarray | Male | 71 | Poorly differentiated | T4a |
| 7 | microarray | Female | 55 | Poorly differentiated | T4b |
| 8 | microarray | Male | 53 | Moderately differentiated | T4a |
| 9 | microarray | Male | 55 | Moderately differentiated | T3 |
| 10 | microarray | Male | 51 | Moderately differentiated | T4a |
| 11 | microarray | Male | 56 | Poorly differentiated | T4a |
| 12 | microarray | Male | 73 | Moderately differentiated | T3 |
| 13 | microarray | Female | 69 | Poorly differentiated | T3 |
| 14 | microarray | Female | 65 | Poorly differentiated | T2 |
| 15 | microarray | Male | 50 | Moderately differentiated | T2 |
| 16 | microarray | Male | 59 | Moderately differentiated | T1b |
| 17 | microarray | Male | 75 | Poorly differentiated | T2 |
| 18 | microarray | Male | 40 | Poorly differentiated | T3 |
| 19 | microarray | Male | 41 | Highly differentiated | T1b |
| 20 | microarray | Female | 76 | Poorly differentiated | T2 |
| 21 | microarray | Female | 51 | Poorly differentiated | T2 |
| 22 | microarray | Male | 36 | Highly differentiated | T2 |
| 23 | microarray | Male | 68 | Highly differentiated | T2 |
| 24 | microarray | Male | 59 | Highly differentiated | T3 |
| 25 | microarray | Male | 74 | Highly differentiated | T1b |
| 26 | microarray | Female | 65 | Highly differentiated | T1b |
| 27 | microarray | Male | 49 | Highly differentiated | T2 |
| 28 | microarray | Male | 58 | Moderately differentiated | T1b |
| 29 | microarray | Male | 70 | Poorly differentiated | T3 |
| 30 | microarray | Female | 56 | Highly differentiated | T3 |
| 31 | microarray | Male | 81 | Moderately differentiated | T3 |
| 32 | microarray | Male | 65 | Moderately differentiated | T3 |
| 33 | microarray | Male | 50 | Moderately differentiated | T2 |
| 34 | microarray | Male | 47 | Moderately differentiated | T2 |
| 35 | microarray | Female | 69 | Moderately differentiated | T2 |
| 36 | microarray | Male | 42 | Poorly differentiated | T3 |
| 37 | microarray | Male | 42 | Moderately differentiated | T2 |
| 38 | microarray | Female | 41 | Poorly differentiated | T4a |
| 39 | microarray | Male | 64 | Poorly differentiated | T4b |
| 40 | microarray | Male | 53 | Moderately differentiated | T3 |
| 41 | microarray | Female | 71 | Poorly differentiated | T3 |
| 42 | microarray | Female | 57 | Poorly differentiated | T4a |
| 43 | microarray | Male | 55 | Moderately differentiated | T1 |
| 44 | microarray | Female | 53 | Moderately differentiated | T1 |
| 45 | microarray | Male | 84 | Moderately differentiated | T1 |
| 1 | miRNA array | Female | 43 | Highly differentiated | T3 |
| 2 | miRNA array | Female | 60 | Poorly differentiated | T3 |
| 3 | miRNA array | Female | 67 | Poorly differentiated | T4a |
| 4 | miRNA array | Female | 78 | Poorly differentiated | T3 |
| 5 | miRNA array | Male | 66 | Highly differentiated | T1b |
| 6 | miRNA array | Female | 54 | Highly differentiated | T1b |
| 7 | miRNA array | Female | 63 | Moderately differentiated | T2 |
| 8 | miRNA array | Male | 48 | Poorly differentiated | T3 |
| 9 | miRNA array | Male | 56 | Moderately differentiated | T2 |
| 10 | miRNA array | Male | 73 | Moderately differentiated | T2 |
| 11 | miRNA array | Male | 53 | Poorly differentiated | T3 |
| 12 | miRNA array | Female | 49 | Moderately differentiated | T3 |
| 13 | miRNA array | Male | 57 | Poorly differentiated | T4a |
| 14 | miRNA array | Male | 59 | Moderately differentiated | T4a |
| 15 | miRNA array | Male | 60 | Poorly differentiated | T3 |
| 1 | RT-PCR | Male | 42 | Poorly differentiated | T1b |
| 2 | RT-PCR | Male | 60 | Poorly differentiated | T2 |
| 3 | RT-PCR | Male | 47 | Poorly differentiated | T4a |
| 4 | RT-PCR | Female | 62 | Moderately differentiated | T3 |
| 5 | RT-PCR | Male | 62 | Poorly differentiated | T4a |
| 6 | RT-PCR | Male | 69 | Highly differentiated | T3 |
| 7 | RT-PCR | Male | 57 | Moderately differentiated | T3 |
| 8 | RT-PCR | Female | 41 | Highly differentiated | T4a |
| 9 | RT-PCR | Female | 70 | Moderately differentiated | T1b |
| 10 | RT-PCR | Male | 70 | Moderately differentiated | T4a |

Gastric cancer invasion was refer to the International Union against Cancer (UICC) TNM staging system. T1: Tumor invades lamina propria, muscularis mucosae, or submucosa; T2: Tumor invades muscularis propria; T3: Tumor penetrates subserosal connective tissue without invasion of visceral peritoneum or adjacent structures; T4: Tumor invades serosa (visceral peritoneum) or adjacent structures
