# Supplementary material for: Overexpression of E2F mRNAs Associated with Gastric Cancer Progression Identified by the Transcription Factor and miRNA Co-Regulatory Network Analysis
Source: PLoS One. 2015 Feb 3;10(2):e0116979. doi: 10.1371/journal.pone.0116979 (PMC4315469; doi:10.1371/journal.pone.0116979)
Supplement: S4 Table — (DOCX) [file pone.0116979.s004.docx]

**Table S4 105 differentially expressed genes in the TFs-regulatory network**

| **mRNA Accession** | **Gene Symbol** | **Description** | **Fold change** | | **P-Value** |
| --- | --- | --- | --- | --- | --- |
| NM_001254 | CDC6 | cell division cycle 6 homolog (S. cerevisiae) (CDC6), mRNA. | | 3.010 | 0.000 |
| NM_001067 | TOP2A | topoisomerase (DNA) II alpha 170kDa (TOP2A), mRNA. | | 2.931 | 0.000 |
| NM_001827 | CKS2 | CDC28 protein kinase regulatory subunit 2 (CKS2), mRNA. | | 2.649 | 0.000 |
| NM_003254 | TIMP1 | TIMP metallopeptidase inhibitor 1 (TIMP1), mRNA. | | 2.534 | 0.000 |
| NM_001003954 | ANXA13 | annexin A13 (ANXA13), transcript variant 2, mRNA. | | 2.526 | 0.000 |
| NM_031966 | CCNB1 | cyclin B1 (CCNB1), mRNA. | | 2.437 | 0.000 |
| NM_001786 | CDC2 | cell division cycle 2, G1 to S and G2 to M (CDC2), transcript variant 1, mRNA. | | 2.375 | 0.000 |
| NM_012310 | KIF4A | kinesin family member 4A (KIF4A), mRNA. | | 2.364 | 0.000 |
| NM_018685 | ANLN | anillin, actin binding protein (ANLN), mRNA. | | 2.273 | 0.000 |
| NM_003981 | PRC1 | protein regulator of cytokinesis 1 (PRC1), transcript variant 1, mRNA. | | 2.253 | 0.000 |
| NM_001274 | CHEK1 | CHK1 checkpoint homolog (S. pombe) (CHEK1), mRNA. | | 2.211 | 0.000 |
| NM_002639 | SERPINB5 | serpin peptidase inhibitor, clade B (ovalbumin), member 5 (SERPINB5), mRNA. | | 2.197 | 0.000 |
| NM_001071 | TYMS | thymidylate synthetase (TYMS), mRNA. | | 2.176 | 0.000 |
| NM_000584 | IL8 | interleukin 8 (IL8), mRNA. | | 2.117 | 0.000 |
| NM_001238 | CCNE1 | cyclin E1 (CCNE1), transcript variant 1, mRNA. | | 2.080 | 0.000 |
| NM_024017 | HOXB9 | homeobox B9 (HOXB9), mRNA. | | 2.050 | 0.000 |
| NM_005030 | PLK1 | polo-like kinase 1 (Drosophila) (PLK1), mRNA. | | 2.031 | 0.000 |
| NM_002915 | RFC3 | replication factor C (activator 1) 3, 38kDa (RFC3), transcript variant 1, mRNA. | | 1.992 | 0.000 |
| NM_203394 | E2F7 | E2F transcription factor 7 (E2F7), mRNA. | | 1.983 | 0.000 |
| NM_002354 | TACSTD1 | tumor-associated calcium signal transducer 1 (TACSTD1), mRNA. | | 1.970 | 0.000 |
| NM_198175 | NME1 | non-metastatic cells 1, protein (NM23A) expressed in (NME1), transcript variant 1, mRNA. | | 1.967 | 0.000 |
| NM_003318 | TTK | TTK protein kinase (TTK), mRNA. | | 1.960 | 0.000 |
| NM_002658 | PLAU | plasminogen activator, urokinase (PLAU), mRNA. | | 1.959 | 0.000 |
| NM_032485 | MCM8 | minichromosome maintenance complex component 8 (MCM8), transcript variant 1, mRNA. | | 1.945 | 0.000 |
| NM_000059 | BRCA2 | breast cancer 2, early onset (BRCA2), mRNA. | | 1.886 | 0.000 |
| NM_001949 | E2F3 | E2F transcription factor 3 (E2F3), mRNA. | | 1.879 | 0.000 |
| NM_007295 | BRCA1 | breast cancer 1, early onset (BRCA1), transcript variant BRCA1b, mRNA. | | 1.870 | 0.000 |
| NM_001081640 | PRKDC | protein kinase, DNA-activated, catalytic polypeptide (PRKDC), transcript variant 2, mRNA. | | 1.856 | 0.000 |
| NM_012484 | HMMR | hyaluronan-mediated motility receptor (RHAMM) (HMMR), transcript variant 1, mRNA. | | 1.846 | 0.000 |
| NM_001037540 | SCML1 | sex comb on midleg-like 1 (Drosophila) (SCML1), transcript variant 1, mRNA. | | 1.830 | 0.000 |
| NM_004219 | PTTG1 | pituitary tumor-transforming 1 (PTTG1), mRNA. | | 1.816 | 0.000 |
| NM_003504 | CDC45L | CDC45 cell division cycle 45-like (S. cerevisiae) (CDC45L), mRNA. | | 1.810 | 0.000 |
| NM_004995 | MMP14 | matrix metallopeptidase 14 (membrane-inserted) (MMP14), mRNA. | | 1.801 | 0.000 |
| NM_002875 | RAD51 | RAD51 homolog (RecA homolog, E. coli) (RAD51), transcript variant 1, mRNA. | | 1.800 | 0.000 |
| NM_004864 | GDF15 | growth differentiation factor 15 (GDF15), mRNA. | | 1.779 | 0.000 |
| NM_004111 | FEN1 | flap structure-specific endonuclease 1 (FEN1), mRNA. | | 1.770 | 0.000 |
| NM_001168 | BIRC5 | baculoviral IAP repeat-containing 5 (survivin) (BIRC5), transcript variant 1, mRNA. | | 1.744 | 0.000 |
| NM_001316 | CSE1L | CSE1 chromosome segregation 1-like (yeast) (CSE1L), mRNA. | | 1.738 | 0.000 |
| NM_031299 | CDCA3 | cell division cycle associated 3 (CDCA3), mRNA. | | 1.713 | 0.000 |
| NM_001845 | COL4A1 | collagen, type IV, alpha 1 (COL4A1), mRNA. | | 1.699 | 0.000 |
| NM_001904 | CTNNB1 | catenin (cadherin-associated protein), beta 1, 88kDa (CTNNB1), mRNA. | | 1.698 | 0.000 |
| NM_004530 | MMP2 | matrix metallopeptidase 2 (MMP2), mRNA. | | 1.696 | 0.000 |
| NM_000417 | IL2RA | interleukin 2 receptor, alpha (IL2RA), mRNA. | | 1.693 | 0.000 |
| NM_000546 | TP53 | tumor protein p53 (Li-Fraumeni syndrome) (TP53), mRNA. | | 1.687 | 0.000 |
| NM_004725 | BUB3 | BUB3 budding uninhibited by benzimidazoles 3 homolog, transcript variant 1, mRNA. | | 1.686 | 0.000 |
| NM_203401 | STMN1 | stathmin 1/oncoprotein 18 (STMN1), transcript variant 1, mRNA. | | 1.673 | 0.000 |
| NM_005342 | HMGB3 | high-mobility group box 3 (HMGB3), mRNA. | | 1.670 | 0.000 |
| NM_175065 | HIST2H2AB | histone cluster 2, H2ab (HIST2H2AB), mRNA. | | 1.670 | 0.000 |
| NM_000251 | MSH2 | mutS homolog 2, colon cancer, nonpolyposis type 1 (E. coli) (MSH2), mRNA. | | 1.661 | 0.000 |
| NM_002009 | FGF7 | fibroblast growth factor 7 (keratinocyte growth factor) (FGF7), mRNA. | | 1.654 | 0.001 |
| NM_003821 | RIPK2 | receptor-interacting serine-threonine kinase 2 (RIPK2), mRNA. | | 1.650 | 0.000 |
| NM_003222 | TFAP2C | transcription factor AP-2 gamma (TFAP2C), mRNA. | | 1.646 | 0.000 |
| NM_004456 | EZH2 | enhancer of zeste homolog 2 (Drosophila) (EZH2), transcript variant 1, mRNA. | | 1.644 | 0.000 |
| NM_007315 | STAT1 | signal transducer and activator of transcription 1, (STAT1), transcript variant alpha, mRNA. | | 1.643 | 0.000 |
| NM_001798 | CDK2 | cyclin-dependent kinase 2 (CDK2), transcript variant 1, mRNA. | | 1.640 | 0.000 |
| NM_000874 | IFNAR2 | interferon (alpha, beta and omega) receptor 2 (IFNAR2), transcript variant 2, mRNA. | | 1.627 | 0.000 |
| NM_018837 | SULF2 | sulfatase 2 (SULF2), transcript variant 1, mRNA. | | 1.621 | 0.000 |
| NM_003414 | ZNF267 | zinc finger protein 267 (ZNF267), transcript variant 498723, mRNA. | | 1.616 | 0.001 |
| NM_002789 | PSMA4 | proteasome (prosome, macropain) subunit, alpha type, 4 (PSMA4), mRNA. | | 1.603 | 0.001 |
| NM_000057 | BLM | Bloom syndrome (BLM), mRNA. | | 1.601 | 0.000 |
| NM_019591 | ZNF26 | zinc finger protein 26 (ZNF26), mRNA. | | 1.600 | 0.000 |
| NM_006739 | MCM5 | minichromosome maintenance complex component 5 (MCM5), mRNA. | | 1.595 | 0.000 |
| NM_002593 | PCOLCE | procollagen C-endopeptidase enhancer (PCOLCE), mRNA. | | 1.595 | 0.000 |
| NM_000321 | RB1 | retinoblastoma 1 (including osteosarcoma) (RB1), mRNA. | | 1.586 | 0.000 |
| NM_001950 | E2F4 | E2F transcription factor 4, p107/p130-binding (E2F4), mRNA. | | 1.573 | 0.000 |
| NM_005915 | MCM6 | minichromosome maintenance complex component 6 (MCM6), mRNA. | | 1.571 | 0.000 |
| NM_002535 | OAS2 | 2'-5'-oligoadenylate synthetase 2, 69/71kDa (OAS2), transcript variant 2, mRNA. | | 1.570 | 0.001 |
| NM_000693 | ALDH1A3 | aldehyde dehydrogenase 1 family, member A3 (ALDH1A3), mRNA. | | 1.561 | 0.000 |
| NM_001909 | CTSD | cathepsin D (CTSD), mRNA. | | 1.560 | 0.000 |
| NM_022121 | PERP | PERP, TP53 apoptosis effector (PERP), mRNA. | | 1.550 | 0.003 |
| AL512709 | TBC1D16 | mRNA; cDNA DKFZp762G216 (from clone DKFZp762G216). | | 1.550 | 0.000 |
| NM_002916 | RFC4 | replication factor C (activator 1) 4, 37kDa (RFC4), transcript variant 1, mRNA. | | 1.550 | 0.000 |
| NM_004859 | CLTC | clathrin, heavy chain (Hc) (CLTC), mRNA. | | 1.548 | 0.000 |
| NM_002497 | NEK2 | NIMA (never in mitosis gene a)-related kinase 2 (NEK2), mRNA. | | 1.546 | 0.000 |
| NM_005381 | NCL | nucleolin (NCL), mRNA. | | 1.545 | 0.000 |
| NM_002703 | PPAT | phosphoribosyl pyrophosphate amidotransferase (PPAT), mRNA. | | 1.543 | 0.000 |
| NM_001033 | RRM1 | ribonucleotide reductase M1 polypeptide (RRM1), mRNA. | | 1.542 | 0.000 |
| NM_005531 | IFI16 | interferon, gamma-inducible protein 16 (IFI16), mRNA. | | 1.541 | 0.001 |
| NM_002388 | MCM3 | minichromosome maintenance complex component 3 (MCM3), mRNA. | | 1.540 | 0.000 |
| NM_003258 | TK1 | thymidine kinase 1, soluble (TK1), mRNA. | | 1.539 | 0.000 |
| NM_001032283 | TMPO | thymopoietin (TMPO), transcript variant 2, mRNA. | | 1.537 | 0.000 |
| NM_003842 | TNFRSF10B | tumor necrosis factor receptor superfamily, (TNFRSF10B), transcript variant 1, mRNA. | | 1.537 | 0.000 |
| NM_006622 | PLK2 | polo-like kinase 2 (Drosophila) (PLK2), mRNA. | | 1.536 | 0.000 |
| NM_032208 | ANTXR1 | anthrax toxin receptor 1 (ANTXR1), transcript variant 1, mRNA. | | 1.533 | 0.000 |
| NM_006466 | POLR3F | polymerase (RNA) III (DNA directed) polypeptide F, 39 kDa (POLR3F), mRNA. | | 1.530 | 0.000 |
| NM_004881 | TP53I3 | tumor protein p53 inducible protein 3 (TP53I3), transcript variant 1, mRNA. | | 1.521 | 0.000 |
| NM_001813 | CENPE | centromere protein E, 312kDa (CENPE), mRNA. | | 1.518 | 0.000 |
| NM_002296 | LBR | lamin B receptor (LBR), transcript variant 1, mRNA. | | 1.515 | 0.002 |
| NM_005225 | E2F1 | E2F transcription factor 1 (E2F1), mRNA. | | 1.515 | 0.000 |
| NM_002415 | MIF | macrophage migration inhibitory factor (glycosylation-inhibiting factor) (MIF), mRNA. | | 1.514 | 0.000 |
| NM_000014 | A2M | alpha-2-macroglobulin (A2M), mRNA. | | 1.513 | 0.001 |
| NM_139276 | STAT3 | signal transducer and activator of transcription 3 (STAT3), transcript variant 1, mRNA. | | 1.511 | 0.000 |
| NM_003579 | RAD54L | RAD54-like (S. cerevisiae) (RAD54L), mRNA. | | 1.509 | 0.000 |
| NM_014288 | ITGB3BP | integrin beta 3 binding protein (beta3-endonexin) (ITGB3BP), mRNA. | | 1.508 | 0.004 |
| NM_002358 | MAD2L1 | MAD2 mitotic arrest deficient-like 1 (yeast) (MAD2L1), mRNA. | | 1.506 | 0.001 |
| NM_003234 | TFRC | transferrin receptor (p90, CD71) (TFRC), mRNA. | | 1.505 | 0.002 |
| NM_001083588 | E2F5 | E2F transcription factor 5, p130-binding (E2F5), transcript variant 2, mRNA. | | 1.504 | 0.000 |
| NM_004217 | AURKB | aurora kinase B (AURKB), mRNA. | | 1.504 | 0.000 |
| NM_003213 | TEAD4 | TEA domain family member 4 (TEAD4), transcript variant 1, mRNA. | | 1.503 | 0.000 |
| NM_004091 | E2F2 | E2F transcription factor 2 (E2F2), mRNA. | | 1.500 | 0.000 |
| NM_139072 | DNER | delta/notch-like EGF repeat containing (DNER), mRNA. | | -1.639 | 0.000 |
| NM_001077243 | GRIA4 | glutamate receptor, ionotrophic, AMPA 4 (GRIA4), transcript variant 2, mRNA. | | -1.854 | 0.000 |
| NM_052832 | SLC26A7 | solute carrier family 26, member 7 (SLC26A7), transcript variant 1, mRNA. | | -2.070 | 0.000 |
| NM_001275 | CHGA | chromogranin A (parathyroid secretory protein 1) (CHGA), mRNA. | | -2.242 | 0.000 |
| NM_170742 | KCNJ16 | potassium inwardly-rectifying channel, subfamily J, member 16 (KCNJ16), transcript variant 3, mRNA. | | -2.243 | 0.000 |
